# Predictive model of 4‐year cognitive decline risk in middle‐aged adults in rural area of Xi'an

**DOI:** 10.1002/alz70860_099020

**Published:** 2025-12-23

**Authors:** Ling Gao, Qiumin Qu

**Affiliations:** ^1^ The First Affiliated Hospital of Xi'an Jiaotong University, Xi'an, Shaanxi, China

## Abstract

**Background:**

To develop risk predictive model of cognitive decline in a prospective cohort study in rural area of Xi’an, and compare the predictive performance with classical CAIDE model.

**Method:**

The cohort was established from October 2014 to March 2015 in two selected villages in rural Xi’an. Mini‐mental State Examination (MMSE) was applied to assess global cognition at baseline and 4‐year follow‐up, and cognitive decline was defined as a drop of ≥4 points in MMSE after 4‐years follow‐up. Participants were randomly split into training set and validation set in a ratio of 7:3, and the logistic regression analysis was used to develop the prediction model, and the area under the receiver operating characteristic curve (AUC) was applied to assess the performance of risk model.

**Result:**

Occurrence of cognitive decline after 4‐years follow‐up was 4.15%. Future cognitive decline was significantly predicted by age, low education and stroke (AUC in training set =0.73; 95% CI:0.63‐0.79; AUC in valid data=0.77; 95% CI:0.67‐0.87), while the classical CAIDE model did not predict risk of cognitive decline well (AUC 0.68; 95% CI:0.61‐0.75). The results differed after stratification by *APOE* genotype, and showed a better predictive value of both our model (AUC 0.87;95%CI:0.78‐0.96) and CAIDE model (AUC 0.89;95%CI:0.81‐0.98) in *APOE* ε4 carriers.

**Conclusion:**

The predictive model was developed based on age, educational level and stroke, and it predicted relatively well of 4‐year cognitive decline as compared with traditional CAIDE model, especially in *APOE* ε4 carriers, but the model should be validated after longer follow‐up and further improved to increase its predictive value.

